# The Development of Generosity From 4 to 6 Years: Examining Stability and the Biopsychosocial Contributions of Children’s Vagal Flexibility and Mothers’ Compassion

**DOI:** 10.3389/fpsyg.2020.590384

**Published:** 2020-11-05

**Authors:** Jonas G. Miller, Sarah Kahle, Natalie R. Troxel, Paul D. Hastings

**Affiliations:** ^1^Department of Psychiatry and Behavioral Sciences, Stanford University, Stanford, CA, United States; ^2^Department of Psychiatry and Behavioral Sciences, University of California, Davis, Sacramento, CA, United States; ^3^Department of Psychology, University of California, Davis, Davis, CA, United States

**Keywords:** altruism, childhood, compassion, polyvagal theory, vagal regulation

## Abstract

Individual differences in children’s prosocial behaviors, including their willingness to give up something of value for the benefit of others, are rooted in physiological and environmental processes. In a sample of 4-year-old children, we previously found evidence that flexible changes in respiratory sinus arrhythmia (RSA) were linked to donation behavior, and that these physiological patterns may support greater sensitivity to the positive effects of compassionate parenting on donation behavior. The current study focused on a follow-up assessment of these children at age 6. First, we examined the stability of individual differences in donation behavior and related parasympathetic nervous system (PNS) activity from age 4 to 6. Second, we examined associations between donation behavior and RSA at 6 years. Third, we examined whether the association between children’s RSA and donation behavior at age 6 varied depending on mothers’ compassionate love. We found low to modest stability in donation behavior and RSA reactivity from age 4 to 6. These findings provide preliminary evidence that stable individual differences in altruism, as reflected by generosity, and in some aspects of parasympathetic functioning during opportunities to be prosocial, emerge in childhood. In addition, we found that some of the same associations between donation behavior, RSA, and compassionate love that we previously observed in children at 4 years of age continued to be evident 2 years later at age 6. Greater decreases in RSA when given the opportunity to donate were associated with children donating more of their own resources which, in turn, were associated with greater RSA recovery after the task. Lastly, mothers’ compassionate love was positively associated with donation behavior in children who demonstrated stronger decreases in RSA during the task; compassionate parenting and RSA reactivity may serve as external and internal supports for prosociality that build on each other. Taken together, these findings contribute to the perspectives that individual differences in altruistic behaviors are intrinsically linked to healthy vagal flexibility, and that biopsychosocial approaches provide a useful framework for examining and understanding the environmental and physiological processes underlying these individual differences.

## Introduction

Young children vary in their propensity for prosocial behaviors, including altruistic actions that require sacrificing something of personal value for the benefit of others. Engaging in prosocial behaviors may confer a number of benefits for young children, including positive emotions (Aknin et al., 2018), healthy friendships (Clark and Ladd, 2000), and protection against the development of early behavioral problems (Hastings et al., 2000). Children who are more prosocial than their peers in early childhood are likely to remain relatively more prosocial later in childhood (Grusec et al., 2011). Nevertheless, this longitudinal stability of individual differences appears to be modest (Eisenberg et al., 2015; Schachner et al., 2018) and could vary for different types of prosocial behaviors. In particular, few studies have considered whether individual differences in children’s donation behavior are stable over time. Determining the stability of donation behaviors in childhood is important for assessing whether efforts to promote early generosity could plausibly have longer-term implications for proneness to altruism later in life.

Physiological regulation of emotion likely supports children’s engagement in and subsequent development of prosocial behaviors (Porges, 1998; Hastings and Miller, 2014). Parasympathetic regulation of cardiovascular activity *via* the myelinated vagus nerve may facilitate social engagement and affective behaviors (Porges, 2011). Decreasing parasympathetic nervous system (PNS) activity in response to challenge can increase cardiovascular activity in support of adaptive orienting and coping, whereas increasing or maintaining PNS activity in safe social contexts may help downregulate stress response systems in favor of a calm, soothed physiological state (Porges, 2011). Given that attention, emotion regulation, and social engagement are all considered important for empathy and prosocial behavior, PNS functioning [as assessed by measures of heart rate variability such as respiratory sinus arrhythmia (RSA)] has garnered significant interest as a physiological correlate of children’s prosociality (Eisenberg et al., 1991; Hastings et al., 2000; Miller et al., 2016, 2017; Acland et al., 2019). For example, Miller et al. (2016) found that young children demonstrated initial decreases followed by increases in PNS activity in response to an empathy induction video, potentially reflecting initial orientation to others’ distress followed by calm social engagement. Children who demonstrated stronger decreases and increases in PNS activity were more likely to report feeling empathic sadness during the video and were more likely to demonstrate prosocial behaviors 2 years later. In addition to this research on PNS regulation and individual differences in prosociality, some studies imply that the act of helping others in and of itself may also be a behavioral strategy for regulating emotion and physiology (Miller et al., 2015; Inagaki and Eisenberger, 2016; Raposa et al., 2016). For example, in a study of adults, Inagaki and Eisenberger (2016) found experimental evidence that participating in a prosocial task resulted in decreased sympathetic nervous system activation in response to a standardized social stress test. Thus, helping others may downregulate stress response systems.

We combined these research perspectives in a previous study with preschool-age children involving a situation in which they could choose to donate earned resources to other children in need (Miller et al., 2015). We found that greater PNS activation (i.e., higher RSA) while listening to an examiner describe the opportunity to donate, potentially indicating calm attentiveness (Porges, 2007; Hastings et al., 2008), was associated with subsequently making greater donations. When given the opportunity to act, decreasing PNS activity (i.e., decreasing RSA), potentially reflecting allocation of energy to orient upon and engage with a task (Calkins, 1997), was associated with donating more. Giving away more earned resources, in turn, was related to stronger PNS recovery as evidenced by RSA increases after donating was completed. Taken together, these findings suggest both that physiological regulation is important for mobilization of prosocial action and that prosocial behavior itself may buffer against prolonged physiological activation. The observed changes in the associations between RSA and donation behavior also suggested that vagal flexibility – the ability to increase and decrease PNS activity as conditions change (Miller et al., 2013) – may play a role in supporting young children’s prosocial behavior.

Although these findings reinforced research suggesting that vagal flexibility is involved in children’s prosociality (Hastings and Miller, 2014; Miller et al., 2016; Miller, 2018), there are still significant gaps in our understanding of the development of such associations. For example, it is unclear whether the links between vagal flexibility and children’s donation behavior observed in Miller et al. (2015) are specific to the preschool-age period or whether they extend into childhood. In addition, we know little about the longitudinal stability of PNS functioning in prosocial contexts. Although prior longitudinal studies have found modest stability of individual differences in PNS responses to cognitive and negative emotional challenges (Calkins and Keane, 2004; Dollar et al., 2020), to date, researchers have not investigated the stability of physiological regulation specific to opportunities to donate or share with others. Thus, the primary aims of this study were to determine whether the associations between vagal flexibility and generosity observed in preschoolers would be maintained in childhood, and to examine the prospective biobehavioral development of associations between vagal flexibility and generosity over this period.

Young children’s prosocial tendencies are not solely a product of individual differences in physiological processes; socialization influences including compassionate caregiving also make important contributions to children’s prosocial tendencies (Hastings et al., 2007, 2015; Brownell, 2016). Parents can model prosocial behaviors and empathy (Eisenberg et al., 2006), socialize prosocial values (Kärtner et al., 2010), and express warmth, sensitivity, and responsiveness to their own children’s needs (Davidov and Grusec, 2006). Some parents attempt to model generosity to their children which can, in turn, lead to children emulating these behaviors (Ben-Ner et al., 2017). Nevertheless, children may vary in their sensitivity to the effects of different caregiving experiences on their prosocial development (Miller and Hastings, 2016, 2019). Several researchers have posited that individual differences in sensitivity to environmental influence are rooted in the functioning of neurobiological systems important for regulating arousal, including the PNS (Del Giudice et al., 2011; Ellis et al., 2011). In addition to mobilizing bodily resources in response to environmental stimuli, PNS functioning may play a role in filtering and encoding environmental information (Del Giudice et al., 2011). Thus, PNS functioning may moderate caregiving effects. Few studies, however, have considered biopsychosocial models of prosocial development that examine the joint, interactive contributions of parenting characteristics and children’s PNS functioning (Miller and Hastings, 2019). Obradović et al. (2010) found that family adversity was negatively associated with young children’s prosocial behaviors (as assessed by self-, mother-, and teacher-report questionnaires) when children showed stronger decreases in PNS activity in response to challenging tasks. Conversely, in a study of maternal emotion socialization strategies and child PNS response to disappointment, Scrimgeour et al. (2016) found that maternal behaviors that emphasized children’s focus on their own negative emotions were associated with less mother-reported prosocial development, but only among children who showed weaker decreases in PNS activity in response to disappointment. The findings from Obradović et al. (2010) and Scrimgeour et al. (2016) both suggest that PNS reactivity may represent vulnerability to the adverse effects of negative family environments on prosocial behavior. The findings from these two studies differ, however, in whether this vulnerability is reflected in increased or decreased PNS reactivity. Other studies have found evidence that PNS functioning indicates susceptibility to environmental influence on prosocial behavior rather than just vulnerability to adverse experiences. For example, a recent study of college students found that self-reported peer and parental attachments were positively associated with self-reported prosocial tendencies among participants with lower RSA during a resting baseline task (Zhang et al., 2020). Individuals with lower resting RSA reported the highest and lowest levels of prosocial behaviors under conditions of high and low attachment security, respectively.

These studies are important contributions to biopsychosocial models of prosocial development, but there are several limitations that are also worth noting (Miller and Hastings, 2019). First, studies have not focused on compassionate caregiving practices that are posited to be important for healthy parent-child relationships implicated in children’s own prosocial development (Grusec et al., 2000; Mikulincer and Shaver, 2005; Hastings et al., 2015). Second, prior studies have primarily focused on children’s PNS activity at rest or in response to challenging or stressful contexts rather than tasks that provide children with opportunities to experience prosocial emotions and to engage in prosocial behaviors (e.g., Eisenberg et al., 1991; Miller et al., 2015, 2016; Coulombe et al., 2019). Third, the existing research on biopsychosocial models of prosocial development has relied almost completely on questionnaire measures of prosocial behaviors and development.

We attempted to address these limitations in further analyses (Miller and Hastings, 2016) of the donation behavior of the 4-year-old children described previously (Miller et al., 2015). In these analyses, we focused on warm, positive aspects of caregiving by assessing mothers’ compassionate love, or their cognitions, attitudes, emotions, and behaviors reflecting a deep sense of love and selfless concern for their child and others (Underwood, 2009). Specifically, we examined whether the association of mothers’ reported compassionate love and the size of children’s donations depended on the children’s RSA while the examiner explained the donation task and while donating. Indeed, at 4 years, more maternal compassionate love predicted greater generosity only for children who demonstrated more vagal flexibility during the altruistic donation task, as reflected in higher RSA during the explanation phase and lower RSA during the active donation phase of the task. Thus, vagal flexibility and compassionate caregiving may serve as sources of children’s prosociality that build on each other. A third aim of the current study was to test whether these findings, observed in preschoolers, would be replicated at a follow-up assessment in childhood.

Here, we report on analyses of the sample of 4-year-old children described in Miller et al. (2015, 2016) who were subsequently invited back to the laboratory 2 years later at 6 years of age. Our first aim was to examine stability of individual differences in donation behavior and vagal flexibility from 4 to 6 years, hypothesizing that both behavior and physiology would evidence significant stability. Second, we examined concurrent associations between RSA and donation behavior at 6 years, hypothesizing that we would replicate the biobehavioral associations observed when children were 4 years old. Third, we examined whether the interactive effect of mothers’ compassionate love and children’s RSA predicted donation behavior, hypothesizing that the links between maternal compassionate love and donation behavior would be stronger for children who showed greater vagal flexibility.

## Materials and Methods

### Participants

The study included 74 preschool-age children at Time 1 (mean age = 4.09 years, *SD* = 0.12; 40 girls, 34 boys). Children were predominantly White (74%) and from middle to upper-middle class families (mean annual family income range = $75,000–$90,000; overall annual income range = $15,000–$30,000 to >$120,000). Children with serious cognitive or physical challenges that might interfere with their ability to understand or complete procedures were excluded from the study. A follow-up assessment took place 2 years later and included 54 mother-child dyads for the current analyses (mean child age = 6.18 years, *SD* = 0.21; 26 boys, 28 girls).

### Procedure

Children were tested in our laboratory using a similar protocol at both time points. Many of the study procedures have been published in Miller et al. (2015). We repeat them here for the sake of reader convenience. After arriving at our laboratory, children played with an examiner for 10 min. During this time, the examiner explained to children that they would be earning tokens over the course of a visit that could be traded in for a prize at the end. Approximately 15 min later, electrodes were attached to the child’s torso to obtain electrocardiograph (ECG) data. After completing a variety of activities over the course of the lab visit, each child gradually earned 20 prize tokens, which were kept for the child in a token box. Just before the end of the visit, the children were presented with an opportunity to donate their prize tokens.

### Measures

#### Donation Behavior at Time 1 and Time 2

At both time points, we administered a donation task (Grusec and Redler, 1980; Miller et al., 2015) at the end of the lab visit before children received their prize. As described in Miller et al. (2015), at Time 1, children were given an opportunity to donate their prize token to anonymous sick children (fictitious), so that the sick children could also get prizes even though they were unable to come into the lab. At Time 2, children were given an opportunity to donate their prize tokens to anonymous children (fictitious) experiencing hardships. At both time points, the task was divided into three phases.

##### Instruction Phase

The examiner sat with the children at a table. Children were told that they had earned 20 prize tokens, enough to get a great prize. The examiner then said that they had another job working with children who were unable to come into the lab to earn prizes. The examiner explained to the child that if they wanted to, they could donate some, none, or all of their tokens by moving tokens from their own box to a separate box for the other children. To check that children understood the task, the examiner asked children to point to their own box and the box for the other children. Children were then given a bell to ring when they were done, and the examiner left the room.

##### Decision Phase

The children were left alone to decide whether and how much to donate by taking tokens out of their own box and placing them into the box for the other children. Children rang the bell when they were done.

##### Conclusion Phase

The examiner returned to the room, closed the boxes without looking inside them, and put away the task materials. The children were not offered feedback on their behavior during this time.

#### Respiratory Sinus Arrhythmia at Time 1 and Time 2

Electrodes were attached to children’s two lower ribs and on the collarbone to collect ECG data using ambulatory monitors. ECG data were sampled at 500 Hz and were wirelessly transmitted to computers for editing and processing using the Heart Rate Variability software from MindWare Technologies (Gahanna, OH, United States). We computed RSA from the ECG data using spectral analysis. The high-frequency bandpass ranged from 0.24 to 1.04 Hz (Huffman et al., 1998). RSA values were computed in 15-s epochs over the course of the donation task. Previous developmental studies have used similar epoch lengths for computing RSA (Huffman et al., 1998; Miller et al., 2013, 2016). RSA values for each epoch within a phase of the task were averaged together. We computed arithmetic difference scores to represent RSA change from one phase of the donation task to the next (e.g., RSA during decision phase minus RSA during introduction phase). Positive and negative RSA change values represented increases and decreases in RSA, respectively.

#### Maternal Compassionate Love at Time 2

Mothers completed the compassionate love scale for humanity and the compassionate love scale for close others to report on their compassionate love for strangers (e.g., “I tend to feel compassion for people, even though I do not know them”) and for their child (e.g., “I often have tender feelings toward my child when she/he seems to be in need”), respectively (Sprecher and Fehr, 2005). For both measures, mothers rated 21 items on a seven-point scale. We first averaged item-responses within measures; compassionate love scores were correlated across measures (*r* = 0.34, *p* = 0.012). We averaged scores across the two scales to form an index of overall compassionate love.

### Analyses

**Figure 1:**
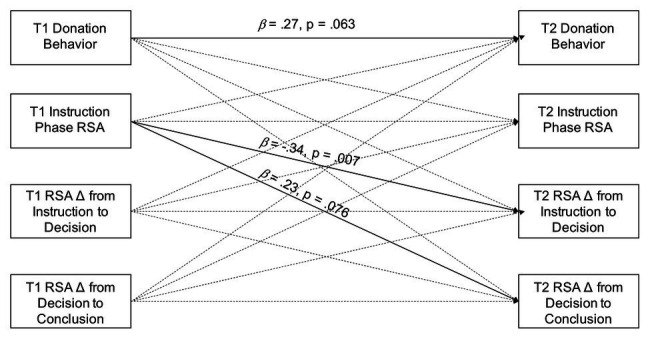
Path model testing of stability of individual differences in donation behavior and RSA from 4 to 6 years of age.

**Figure 2:**
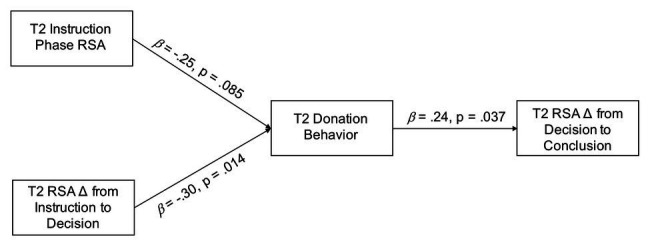
Path model testing cross-sectional associations among donation behavior and RSA at 6 years of age.

We conducted zero-order correlations to examine stability in donation behavior and RSA from 4 to 6 years. We further examined stability of individual differences in a path analysis model that simultaneously included donation behavior, instruction phase RSA, RSA change from instruction to decision, and RSA change from decision to conclusion at age 4 as predictors of these same variables from the age 6 assessment (see Figure 1). In a second path analysis model, we tested concurrent associations between donation behavior and RSA at age 6. This model included instruction phase RSA and RSA change from instruction to decision as predictors of donation behavior which, in turn, was modeled as a predictor of RSA change from decision to conclusion (see Figure 2). The third analysis examined whether maternal compassionate love and RSA interacted to predict donation behavior. This analysis included a path analysis model testing whether maternal compassionate love interacted with instruction phase RSA and with RSA change from instruction to decision to predict donation behavior. Variables were centered prior to forming interaction terms. Statistically significant interactions were probed further by testing simple slopes at 1 SD above and below the mean of the RSA. Gender was not associated with variables of interest and was not included as a covariate in analyses. All models used maximum likelihood estimation to handle missing data. We estimated robust SEs for model parameters. All path analysis models were conducted using the lavaan package (Rosseel, 2012) in R software (R Core Team, 2017).

## Results

Table 1 presents the descriptive statistics and comparisons between Time 1 and Time 2 donation behavior and RSA. On average, children had significantly higher RSA during the instruction phase of the task, and significantly less RSA recovery from the decision to the conclusion phase of the task, at Time 2 compared to Time 1. Children did not show statistically significant differences in donation behavior and RSA change from the instruction to decision phase of the task at Time 1 and Time 2. We also examined zero-order correlations between RSA variables within Time 1 and Time 2. At both time points, higher RSA during the instruction phase was associated with greater RSA suppression to the decision phase of the task (*r* = −0.37, *p* = 0.019 and *r* = −0.41, *p* < 0.001 at Time 1 and Time 2, respectively), and greater RSA suppression to the decision was associated with greater RSA recovery to the conclusion of the task (*r* = −0.56, *p* < 0.001 and *r* = −0.49, *p* < 0.001 at Time 1 and Time 2, respectively). Initial RSA levels at the start of the task were not associated with RSA recovery to the conclusion of the task at either time point (both *p* > 0.83).

**Table 1:** Descriptive statistics, mean comparisons, and zero-order correlations from Time 1 to Time 2.

		Time 1		Time 2		Mean comparison		Correlation	
	Mean	*SD*	Mean	*SD*	*t*-value	*p* value	*r*	*p* value
Tokens given	5.09	6.34	4.10	4.27	1.17	0.249	0.24	0.084
Instruction phase RSA	5.45	1.15	6.44	0.97	6.05	<0.001	0.21	0.246
RSA change from instruction to decision phase	−0.00	0.95	−0.07	0.72	0.82	0.418	0.26	0.035
RSA change from decision to conclusion phase	0.54	0.74	−0.14	0.79	4.24	<0.001	0.14	0.295

RSA, respiratory sinus arrhythmia.

**Table 2:** Regression model testing interactions between maternal compassionate love and child RSA concurrently predicting donation behavior at age 6.

	*B*	*SE*	*β*	*p*
T2 Instruction phase RSA	−1.22	0.61	−0.28	0.044
T2 RSA change from instruction to decision phase	−1.25	0.75	−0.21	0.092
T2 maternal compassionate love	0.32	0.60	0.05	0.591
T2 instruction RSA × compassionate love	1.10	0.79	0.14	0.165
T2 RSA change from instruction to decision × compassionate love	−1.95	0.77	−0.18	0.019

SE, standard error; T2, Time 2; RSA, respiratory sinus arrhythmia.

### Stability of Individual Differences in Donation Behavior and RSA From 4 to 6 Years of Age

Table 1 presents the zero-order correlations testing for stability of individual differences in donation behavior and RSA. There was modest stability in change in RSA from the instruction to decision phase from age 4 to 6 (*r* = 0.26, *p* = 0.035). Children who donated more at age 4 tended to donate more at age 6, but this trend did not reach statistical significance (*r* = 0.24, *p* = 0.084). In addition to stability in RSA during specific phases of the donation task, higher RSA during the instruction phase at 4 years was associated with greater RSA suppression from instruction to decision at 6 years (*r* = −0.36, *p* < 0.001).

Figure 1 presents the path analysis model testing for stability of individual differences in donation behavior and RSA while controlling for all paths from age 4 to 6 variables. Only the association between higher RSA during the instruction phase at 4 years and greater RSA suppression from instruction to decision at 6 years remained statistically significant in the context of controlling for the other variables at age 4 (*β* = −0.34, *p* = 0.007). There were, however, trend-level effects of donation behavior at age 4 predicting donation behavior at age 6 (*β* = 0.27, *p* = 0.063) and RSA during the instruction phase at age 4 predicting RSA recovery at age 6 (*β* = 0.23, *p* = 0.076).

### Concurrent Associations Between Donation Behavior and RSA at 6 Years of Age

Figure 2 presents the path analysis testing for concurrent associations between donation behavior and RSA at age 6. Contrary to what we observed at 4 years (Miller et al., 2015), 6-year-old children’s RSA during the instruction phase was weakly but not significantly associated with donating behavior (*β* = −0.25, *p* = 0.085). Conversely, consistent with our previous findings, greater RSA suppression from instruction to decision was associated with donating more tokens (*β* = −0.30, *p* = 0.014), which, in turn, was associated with greater RSA recovery from decision to conclusion (*β* = 0.24, *p* = 0.037).

### Interactions Involving Maternal Compassionate Love, Child RSA, and Donation Behavior at 6 Years of Age

**Figure 3:**
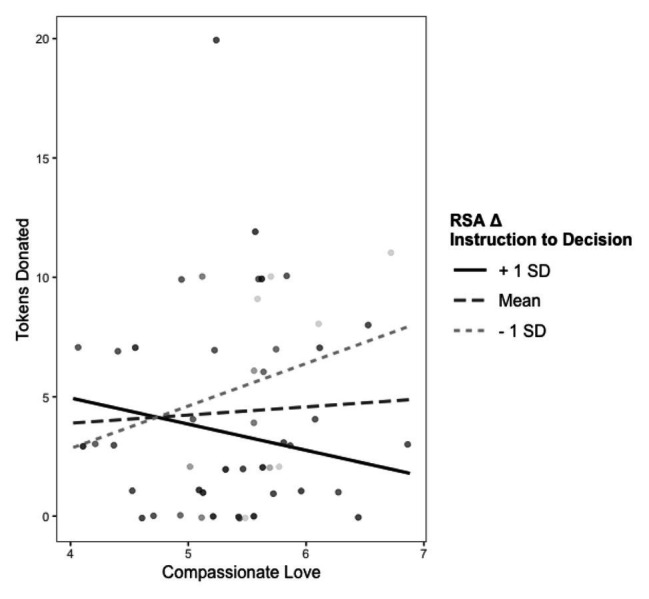
Interaction between maternal compassionate love and RSA change from instruction to decision predicting donation behavior. +1 SD on RSA Δ Instruction to Decision represents increases in RSA from the instruction to decision phase. −1 SD on RSA Δ Instruction to Decision represents stronger decreases in RSA from the instruction to decision phase (i.e., greater RSA suppression).

Table 2 presents the model testing the interactions between child RSA and maternal compassionate love as predictors of donation behavior. Contrary to what we observed at 4 years (Miller and Hastings, 2016), the interaction of compassionate love with child RSA during the instruction phase was not associated with donation behavior (*p* = 0.17). Conversely, and consistent with the earlier observed pattern, the degree to which compassionate love was associated with donation behavior was moderated by RSA suppression from instruction to decision (*β* = −0.18, *p* = 0.012). This interaction is presented in Figure 3. There was a positive association between maternal compassionate love and donation for children who exhibited greater RSA suppression (*β* = 0.30, *p* = 0.022) but not for children who exhibited less RSA suppression (*β* = −0.14, *p* = 0.259).

## Discussion

There are considerable individual differences in young children’s willingness to give up something of value for the benefit of others. Investigating the biobehavioral development of children’s donation behavior is important for understanding these individual differences. We found modest stability in donation behavior and specific components of vagal regulation from age 4 to 6. It should be noted, however, that the longitudinal stability of donation behavior was a borderline effect. In addition to assessing the stability of individual differences in donation behavior and physiology, we examined whether we would observe at 6 years our prior findings with preschool-age children suggesting that vagal flexibility is directly associated with donating more to others (Miller et al., 2015), and that children who demonstrate greater vagal flexibility are even more generous when their mothers report higher levels of compassionate love (Miller and Hastings, 2016). Indeed, some of the same associations between donation behavior, RSA, and compassionate love observed at 4 years continued to be evident 2 years later. Taken together, the current findings contribute to the field’s growing understanding of the biopsychosocial bases of early prosocial development.

To our knowledge, these analyses are among the first to test and provide preliminary evidence for modest stability in individual differences in generosity and related parasympathetic regulation over the course of early childhood. Children who donated more tokens at 4 years also tended to donate more at 6 years, which is consistent with previous studies of other kinds of prosocial behaviors suggesting the emergence of a prosocial disposition in childhood (Eisenberg et al., 2015; Schachner et al., 2018). Anonymous prosocial actions like donating to unfamiliar children may not be as stable from preschool-age to early childhood as is the case for some other forms of prosocial behavior like providing help directly to an individual (Miller et al., 2016), but further research with larger samples is necessary to confirm this finding.

Our study also assessed the stability of PNS functioning, during different phases of the donation task, across time. When examining longitudinal stability using zero-order correlations, we found that children who demonstrated greater RSA suppression from the instruction to the decision phase at 4 years were likely to continue demonstrating this pattern at 6 years. This finding extends previous studies showing that individual differences in PNS responses to cognitive and emotional challenges are modestly stable over time (Calkins and Keane, 2004; Dollar et al., 2020); RSA suppression when presented with an opportunity to act and give to others in need may be a stable individual-difference variable. Conversely, RSA levels during the instruction phase and RSA recovery from the decision to conclusion phases of the task were not stable across time (i.e., not associated from age 4 to 6). It has been noted previously that the stability of RSA may depend on the context in which it is measured (El-Sheikh, 2005). Children’s RSA in the initial stages of learning about others in need of help, and RSA recovery following the conclusion of an opportunity to take prosocial action, may reflect physiological processes at the time of measurement rather than trait-like parasympathetic responding. For example, it is possible that the introduction and conclusions phases of the task elicited different emotions or similar emotions but to different degrees, from children when they were age 4 vs. 6 due to children’s maturation at the second time point. Conversely, the physiological task demands during the decision phase may have been more consistent across assessments. To our knowledge, this is one of the first longitudinal studies of stability of RSA recovery; in separate analyses of this sample of children, we also failed to see longitudinal stability of RSA recovery from frustration (Kahle et al., 2018, under review). Given the potential importance of autonomic recovery processes for children’s emotion and self-regulation (Obradović and Finch, 2017; Kahle et al., 2018; Rudd and Yates, 2018), more research is warranted that considers the stability of RSA recovery across different kinds of tasks.

Our most robust longitudinal association was not an aspect of direct stability. Rather, it was between higher RSA during the instructions phase at 4 years and greater RSA suppression from instruction to decision at 6 years. This association was evident in both the zero-order correlation analysis and path analysis and in cross-sectional analyses at both age 4 and 6. Polyvagal theory (Porges, 2011) posits that higher RSA during safe contexts (e.g., at rest) may indicate capacity for releasing the vagal brake (i.e., RSA suppression) in response to challenge. To our knowledge, the current study is the first to demonstrate that higher RSA in contexts that call for calm social engagement may represent capacity for RSA suppression in response to an opportunity to take prosocial action. RSA suppression from the passive context of the instruction phase of the task to the active context of the decision phase likely supported the mobilization of resources for engaging in the physical act of donating tokens, potentially without the need for engaging the metabolically demanding sympathetic nervous system (Porges, 2011). Consistent with this formulation, we found here that RSA suppression was associated with donating more tokens. The PNS may be part of a social engagement system that is important for positive other-oriented emotions and behaviors (Porges, 2011). Some young children appear to understand and appropriately regulate biobehavioral processes in a prosocial context even in the absence of directly observable cues for helping, such as another person’s expression of suffering or request for help.

Studying this sample at age 4, we previously found that vagal flexibility was associated with children’s donation behavior (Miller et al., 2015). Specifically, higher RSA during the initial instruction phase of the task and greater RSA suppression from the instruction to decision phase, were associated with children donating more tokens, which in turn was associated with greater RSA recovery in the conclusion of the task. The current analyses of the associations among RSA and donation behavior at age 6 replicate all but the first part of the pattern we observed at age 4. Although initial RSA levels during the instruction phase did not predict donation behavior at 6 years, greater RSA suppression continued to predict donating more tokens at 6 years, and donating more tokens predicted greater subsequent RSA recovery. Greater parasympathetic influence during the conclusion phase of the task may support perceptions of safety (conscious or unconscious) and the experience of calmness in a non-challenging context (Thayer and Lane, 2009; Porges, 2011). Other studies have also found that toddlers show decreased autonomic arousal after helping others (Hepach et al., 2012) and that prosocial behavior in adults leads to diminished sympathetic nervous system reactivity to stress (Inagaki and Eisenberger, 2016). The current findings add to this growing body of research suggesting that prosocial behaviors may be intrinsically effective for soothing one’s own arousal (Miller, 2018). Autonomic states underlying perceptions of safety could be one path by which prosocial behaviors confer health benefits across different ages, including childhood. RSA recovery following generous behaviors may also serve as a physiological reinforcement of helping others; some positive emotion states are associated with increased RSA (Kreibig, 2010). Further research, however, is necessary to determine whether RSA recovery supports positive emotions that have been shown promote and reward children’s prosocial behaviors (Aknin et al., 2018).

We did not find evidence for the hypothesized link between higher RSA levels during the initial instructions phase of the task and donating more tokens, which we observed when the children were younger (Miller et al., 2015). In fact, the association was in the opposite direction of what we previously observed (negative instead of positive), albeit not at a statistically significant level. When first learning about an opportunity to donate to other children, greater parasympathetic activation at age 4 may support calm social engagement, allowing for the experience of prosocial emotions such as compassion (Stellar et al., 2015). By age 6, children may have been more readily able to understand the task, such that parasympathetic support for calm attentiveness was less necessary, and withdrawal of parasympathetic influence was initiated sooner in preparation for prosocial action. Another possibility is that at age 6, other physiological processes during the instructions phase of the task could be more important for donation behavior than PNS activity. Further research is needed to understand this inconsistency from age 4 to 6.

We also previously found evidence that vagal flexibility moderates the association between maternal compassionate love and donation behavior. Compassionate love was associated with donating more tokens in preschoolers who demonstrated higher initial RSA and greater RSA suppression to the decision phase of the task (Miller and Hastings, 2016). Conversely, compassionate love was not associated with donation behavior in children who demonstrated less vagal flexibility (i.e., lower initial RSA and less RSA suppression to the decision phase of the task). In the current study, we found partial support for these moderation effects at the follow-up assessment when children were 6 years old. Specifically, we found that maternal compassionate love was positively associated with the number of tokens donated in children who showed greater RSA suppression during the decision phase of the task. This interaction effect suggests that children who demonstrate increased parasympathetic reactivity (i.e., RSA suppression) in prosocial contexts may be particularly sensitive to the positive effects of warm, supportive caregiving from their mothers. Compassionate mothers likely develop emotionally close relationships with their children while also providing an early example of prosocial orientation toward the needs of others. RSA suppression, in addition to coordinating bodily resources for prosocial action, may play a role in encoding environmental information and thus indicate openness to environmental influence (Del Giudice et al., 2011). Taken together, the current study shows the importance of considering children’s biobehavioral coordination as embedded in the socializing context of children’s close relationships. Our finding suggests that at age 6, RSA suppression and compassionate parenting may serve as internal and external supports for the capacity to act prosocially that build on each other. This finding is also consistent with the formulation that certain child features represent exclusive susceptibility to the benefits of supportive family environments (Pluess and Belsky, 2013). This finding, however, differs from previous studies suggesting that parasympathetic functioning may buffer or exacerbate negative parenting effects on children’s prosocial behavior (Obradović et al., 2010; Scrimgeour et al., 2016). Methodological differences may explain these apparent inconsistencies between our finding and those from previous studies. We considered maternal compassionate love, parasympathetic regulation in a prosocial context, and children’s observed donation behavior, whereas prior studies focused on other aspects of the family environment, parasympathetic regulation in non-prosocial contexts, and questionnaire-based measures of prosocial behavior (Obradović et al., 2010; Scrimgeour et al., 2016; Zhang et al., 2020). The differences across study findings also could reflect the nuances of biopsychosocial processes implicated in prosocial development. Increased and decreased parasympathetic reactivity are appropriate in different contexts (Hastings et al., 2008), and different aspects of the caregiving environment may contribute to different aspects of children’s social-emotional development (Grusec and Davidov, 2010). It is possible that specific aspects of caregiving have a greater impact on children’s general positive social development when matched with specific physiological processes in children. Future research is needed to increase our understanding of the complex coordination of context, experience, and physiology that underlies individual differences in prosocial behavior.

We did not, however, observe an interaction between maternal compassionate love and children’s initial RSA levels during the start of the task. One potential explanation for this inconsistency from the earlier analyses at age 4 is that RSA suppression reflects a more stable individual difference variable than initial RSA levels at the start of the task, and this stability could support RSA suppression serving as a moderator of environmental input. Indeed, we observed modest longitudinal stability in RSA reactivity to the decision phase of the task but did not observe significant stability in RSA levels during the instructions phase from age 4 to 6.

There are limitations of our study that should be acknowledged and addressed in future attempt to replicate and extend the findings. First, our small sample size may have limited our ability to detect effects, such as the longitudinal stability of donation behavior, at conventional levels of statistical significance. Second, given our correlational study design, we cannot infer whether RSA suppression plays a causal role in donation behavior or whether donation behavior plays a causal role in RSA recovery. Lastly, we assessed parasympathetic regulation in a donation task, but children’s prosocial behaviors are rooted in the coordination of multiple neurobiological systems (Hastings and Miller, 2014; Miller and Hastings, 2019). Future research that incorporates other physiological measures could help to further illuminate the neurobiological bases of children’s donation behaviors, and help determine whether there are different neurobiological paths to being more generous (Miller, 2018).

The current study represents an important step forward in biobehavioral research on prosocial development. These findings provide preliminary evidence that individual differences in proneness to generosity, and individual differences in some aspects of parasympathetic functioning during these prosocial situations, are emerging in childhood. Our findings also provide an important partial replication of prior findings with this sample at an earlier assessment (Miller et al., 2015; Miller and Hastings, 2016). Conversely, inconsistencies between our current and past findings may be due to developmental differences between 4- and 6-year-old children’s PNS responses to donation opportunities. Taken together, this body of work contributes to the perspectives that individual differences in prosocial behaviors are intrinsically linked to healthy vagal flexibility, and that biopsychosocial approaches provide a useful framework for examining and understanding the environmental and physiological processes underlying these individual differences.

## Data Availability Statement

The raw data supporting the conclusions of this article will be made available by the authors, without undue reservation.

## Ethics Statement

The studies involving human participants were reviewed and approved by University of California, Davis Institutional Review Board. Written informed consent to participate in this study was provided by the participants’ legal guardian/next of kin.

## Author Contributions

All authors designed the study. JM, SK, and NT collected the data. JM processed and analyzed the data and drafted the manuscript. PH provided critical revisions. All authors contributed to the article and approved the submitted version.

### Conflict of Interest

The authors declare that the research was conducted in the absence of any commercial or financial relationships that could be construed as a potential conflict of interest.

## References

[ref1] Acland E. L. Colasante T. Malti T. (2019). Respiratory sinus arrhythmia and prosociality in childhood: evidence for a quadratic effect. Dev. Psychobiol. 61, 1146–1156. 10.1002/dev.21872, PMID: 31206629

[ref2] Aknin L. B. Van de Vondervoort J. W. Hamlin J. K. (2018). Positive feelings reward and promote prosocial behavior. Curr. Opin. Psychol. 20, 55–59. 10.1016/j.copsyc.2017.08.017, PMID: 28837957

[ref3] Ben-Ner A. List J. A. Putterman L. Samek A. (2017). Learned generosity? An artefactual field experiment with parents and their children. J. Econ. Behav. Organ. 143, 28–44. 10.1016/j.jebo.2017.07.030

[ref4] Brownell C. A. (2016). Prosocial behavior in infancy: the role of socialization. Child Dev. Perspect. 10, 222–227. 10.1111/cdep.12189

[ref5] Calkins S. D. (1997). Cardiac vagal tone indices of temperamental reactivity and behavioral regulation in young children. Dev. Psychobiol. 31, 125–135. 10.1002/(sici)1098-2302(199709)31:2<125::aid-dev5>3.0.co;2-m, PMID: 9298638

[ref6] Calkins S. D. Keane S. P. (2004). Cardiac vagal regulation across the preschool period: stability, continuity, and implications for childhood adjustment. Dev. Psychobiol. 45, 101–112. 10.1002/dev.20020, PMID: 15505799

[ref7] Clark K. E. Ladd G. W. (2000). Connectedness and autonomy support in parent-child relationships: links to children’s socioemotional orientation and peer relationships. Dev. Psychol. 36, 485–498. 10.1037/0012-1649.36.4.485, PMID: 10902700

[ref8] Coulombe B. R. Rudd K. L. Yates T. M. (2019). Children’s physiological reactivity in emotion contexts and prosocial behavior. Brain Behav. 9:e01380. 10.1002/brb3.1380, PMID: 31523938 PMC6790335

[ref9] Davidov M. Grusec J. E. (2006). Untangling the links of parental responsiveness to distress and warmth to child outcomes. Child Dev. 77, 44–58. 10.1111/j.1467-8624.2006.00855.x, PMID: 16460524

[ref10] Del Giudice M. Ellis B. J. Shirtcliff E. A. (2011). The adaptive calibration model of stress responsivity. Neurosci. Biobehav. Rev. 35, 1562–1592. 10.1016/j.neubiorev.2010.11.007, PMID: 21145350 PMC3068241

[ref11] Dollar J. M. Calkins S. D. Berry N. T. Perry N. B. Keane S. P. Shanahan L.. (2020). Developmental patterns of respiratory sinus arrhythmia from toddlerhood to adolescence. Dev. Psychol. 56, 783–794. 10.1037/dev0000894, PMID: 31999180 PMC8188730

[ref12] Eisenberg N. Eggum-Wilkens N. D. Spinrad T. L. (2015). “The development of prosocial behavior” in The Oxford handbook of prosocial behavior. eds. Schroeder D. A. Graziano W. G. (Oxford, UK: Oxford University Press), 114–136.

[ref13] Eisenberg N. Fabes R. A. Schaller M. Carlo G. Miller P. A. (1991). The relations of parental characteristics and practices to children’s vicarious emotional responding. Child Dev. 62, 1393–1408. 10.1111/j.1467-8624.1991.tb01613.x, PMID: 1786723

[ref14] Eisenberg N. Fabes R. A. Spinrad T. L. (2006). “Prosocial development” in Handbook of child psychology: Social, emotional, and personality development. 6th Edn. Vol. 3 eds. Damon W. Lerner R. M. Esienberg N. (Hoboken, NJ: John Wiley and Sons, Inc), 646–718.

[ref15] Ellis B. J. Boyce W. T. Belsky J. Bakermans-Kranenburg M. J. van Ijzendoorn M. H. (2011). Differential susceptibility to the environment: an evolutionary—neurodevelopmental theory. Dev. Psychopathol. 23, 7–28. 10.1017/S0954579410000611, PMID: 21262036

[ref16] El-Sheikh M. (2005). Stability of respiratory sinus arrhythmia in children and young adolescents: a longitudinal examination. Dev. Psychobiol. 46, 66–74. 10.1002/dev.20036, PMID: 15690389

[ref17] Grusec J. E. Davidov M. (2010). Integrating different perspectives on socialization theory and research: a domain-specific approach. Child Dev. 81, 687–709. 10.1111/j.1467-8624.2010.01426.x, PMID: 20573097

[ref18] Grusec J. E. Goodnow J. J. Kuczynski L. (2000). New directions in analyses of parenting contributions to children’s acquisition of values. Child Dev. 71, 205–211. 10.1111/1467-8624.00135, PMID: 10836575

[ref900] Grusec J. E. Hastings P. D. Almas A. (2011). “Helping and prosocial behavior” in Handbook of childhood social development. 2nd Edn. eds. Hart C. Smith P. (Malden, MA: Wiley-Blackwell), 549–566. PMID:

[ref19] Grusec J. E. Redler E. (1980). Attribution, reinforcement, and altruism: a developmental analysis. Dev. Psychol. 16, 525–534. 10.1037/0012-1649.16.5.525

[ref20] Hastings P. D. Miller J. G. (2014). “Autonomic regulation, polyvagal theory, and children’s prosocial development” in Prosocial development: A multidimensional approach. eds. Padilla-Walker L. Carlo G. (New York, NY: Oxford University Press), 112–127.

[ref21] Hastings P. D. Miller J. G. Troxel N. R. (2015). “Making good: the socialization of children’s prosocial development” in Handbook of socialization: Theory and research. 2nd Edn. eds. Grusec J. Hastings P. D. (New York, NY: The Guilford Press), 637–660.

[ref22] Hastings P. D. Nuselovici J. N. Utendale W. T. Coutya J. McShane K. E. Sullivan C. (2008). Applying the polyvagal theory to children’s emotion regulation: social context, socialization, and adjustment. Biol. Psychol. 79, 299–306. 10.1016/j.biopsycho.2008.07.005, PMID: 18722499

[ref23] Hastings P. D. Utendale W. T. Sullivan C. (2007). “The socialization of prosocial development” in Handbook of socialization: Theory and research. eds. Grusec J. Hastings P. D. (New York, NY: The Guilford Press), 638–664.

[ref24] Hastings P. D. Zahn-Waxler C. Robinson J. Usher B. Bridges D. (2000). The development of concern for others in children with behavior problems. Dev. Psychol. 36, 531–546. 10.1037/0012-1649.36.5.531, PMID: 10976595

[ref25] Hepach R. Vaish A. Tomasello M. (2012). Young children are intrinsically motivated to see others helped. Psychol. Sci. 23, 967–972. 10.1177/0956797612440571, PMID: 22851443

[ref26] Huffman L. C. Bryan Y. E. Carmen R. del Pedersen F. A. Doussard-Roosevelt J. A. Forges S. W. (1998). Infant temperament and cardiac vagal tone: assessments at twelve weeks of age. Child Dev. 69, 624–635. 10.1111/j.1467-8624.1998.tb06233.x, PMID: 9680676

[ref27] Inagaki T. K. Eisenberger N. I. (2016). Giving support to others reduces sympathetic nervous system-related responses to stress. Psychophysiology 53, 427–435. 10.1111/psyp.12578, PMID: 26575283

[ref28] Kahle S. Miller J. G. Helm J. L. Hastings P. D. (2018). Linking autonomic physiology and emotion regulation in preschoolers: the role of reactivity and recovery. Dev. Psychobiol. 60, 775–788. 10.1002/dev.21746, PMID: 29926898

[ref29] Kärtner J. Keller H. Chaudhary N. (2010). Cognitive and social influences on early prosocial behavior in two sociocultural contexts. Dev. Psychol. 46, 905–914. 10.1037/a0019718, PMID: 20604610

[ref30] Kreibig S. D. (2010). Autonomic nervous system activity in emotion: a review. Biol. Psychol. 84, 394–421. 10.1016/j.biopsycho.2010.03.010, PMID: 20371374

[ref31] Mikulincer M. Shaver P. R. (2005). Attachment security, compassion, and altruism. Curr. Dir. Psychol. Sci. 14, 34–38. 10.1111/j.0963-7214.2005.00330.x

[ref32] Miller J. G. (2018). Physiological mechanisms of prosociality. Curr. Opin. Psychol. 20, 50–54. 10.1016/j.copsyc.2017.08.018, PMID: 28837956

[ref33] Miller J. G. Chocol C. Nuselovici J. N. Utendale W. T. Simard M. Hastings P. D. (2013). Children’s dynamic RSA change during anger and its relations with parenting, temperament, and control of aggression. Biol. Psychol. 92, 417–425. 10.1016/j.biopsycho.2012.12.005, PMID: 23274169 PMC4055035

[ref34] Miller J. G. Hastings P. D. (2016). “Biopsychosocial models of prosociality: compassionate love, vagal regulation, and children’s altruism” in Contexts for young child flourishing: Evolution, family, and society. eds. Narvaez D. Braungart-Rieker J. Miller-Graff L. Gettler L. Hastings P. D. (New York, NY: Oxford University Press), 185–200.

[ref35] Miller J. G. Hastings P. D. (2019). “Parenting, neurobiology, and prosocial development” in The Oxford Handbook of parenting and moral development. eds. Laible D. J. Carlo G. Padilla-Walker L. M. (New York, NY: Oxford University Press).

[ref36] Miller J. G. Kahle S. Hastings P. D. (2015). Roots and benefits of costly giving: children who are more altruistic have greater autonomic flexibility and less family wealth. Psychol. Sci. 26, 1038–1045. 10.1177/0956797615578476, PMID: 26015412 PMC4504814

[ref37] Miller J. G. Kahle S. Hastings P. D. (2017). Moderate baseline vagal tone predicts greater prosociality in children. Dev. Psychol. 53, 274–289. 10.1037/dev0000238, PMID: 27819463 PMC5293607

[ref38] Miller J. G. Nuselovici J. N. Hastings P. D. (2016). Nonrandom acts of kindness: parasympathetic and subjective empathic responses to sadness predict children’s prosociality. Child Dev. 87, 1679–1690. 10.1111/cdev.12629, PMID: 28262932 PMC5340080

[ref39] Obradović J. Bush N. R. Stamperdahl J. Adler N. E. Boyce W. T. (2010). Biological sensitivity to context: the interactive effects of stress reactivity and family adversity on socioemotional behavior and school readiness. Child Dev. 81, 270–289. 10.1111/j.1467-8624.2009.01394.x, PMID: 20331667 PMC2846098

[ref40] Obradović J. Finch J. E. (2017). Linking executive function skills and physiological challenge response: piecewise growth curve modeling. Dev. Sci. 20:e12476. 10.1111/desc.12476, PMID: 27748016

[ref510] Pluess M. Belsky J. (2013). Vantage sensitivity: individual differences in response to positive experiences. Psychol. Bull. 139, 901–916. 10.1037/a0030196, PMID: 23025924

[ref41] Porges S. W. (1998). Love: an emergent property of the mammalian autonomic nervous system. Psychoneuroendocrinology 23, 837–861. 10.1016/S0306-4530(98)00057-2, PMID: 9924740

[ref42] Porges S. W. (2007). The polyvagal perspective. Biol. Psychol. 74, 116–143. 10.1016/j.biopsycho.2006.06.009, PMID: 17049418 PMC1868418

[ref43] Porges S. W. (2011). The polyvagal theory: Neurophysiological foundations of emotions, attachment, communication, and self-regulation. New York, NY: W W Norton and Co., 347.

[ref44] Raposa E. B. Laws H. B. Ansell E. B. (2016). Prosocial behavior mitigates the negative effects of stress in everyday life. Clin. Psychol. Sci. 4, 691–698. 10.1177/2167702615611073, PMID: 27500075 PMC4974016

[ref700] R Core Team (2017). R: A language and environment for statistical computing. R Foundation for Statistical Computing, Vienna, Austria. Available at: https://www.R-project.org

[ref45] Rosseel Y. (2012). Lavaan: an R package for structural equation modeling. J. Stat. Softw. 48, 1–36. 10.18637/jss.v048.i02

[ref46] Rudd K. L. Yates T. M. (2018). The implications of sympathetic and parasympathetic regulatory coordination for understanding child adjustment. Dev. Psychobiol. 60, 1023–1036. 10.1002/dev.21784, PMID: 30370630

[ref47] Schachner A. C. W. Newton E. K. Thompson R. A. Goodman-Wilson M. (2018). Becoming prosocial: the consistency of individual differences in early prosocial behavior. Early Child. Res. Q. 43, 42–51. 10.1016/j.ecresq.2018.01.001

[ref48] Scrimgeour M. B. Davis E. L. Buss K. A. (2016). You get what you get and you don’t throw a fit!: emotion socialization and child physiology jointly predict early prosocial development. Dev. Psychol. 52, 102–116. 10.1037/dev0000071, PMID: 26569566 PMC4695310

[ref49] Sprecher S. Fehr B. (2005). Compassionate love for close others and humanity. J. Soc. Pers. Relat. 22, 629–651. 10.1177/0265407505056439

[ref50] Stellar J. E. Cohen A. Oveis C. Keltner D. (2015). Affective and physiological responses to the suffering of others: compassion and vagal activity. J. Pers. Soc. Psychol. 108, 572–585. 10.1037/pspi0000010, PMID: 25621856

[ref51] Thayer J. F. Lane R. D. (2009). Claude Bernard and the heart–brain connection: further elaboration of a model of neurovisceral integration. Neurosci. Biobehav. Rev. 33, 81–88. 10.1016/j.neubiorev.2008.08.004, PMID: 18771686

[ref52] Underwood L. G. (2009). “Compassionate love: a framework for research” in The science of compassionate love. eds. Fehr B. Sprecher S. Underwood L. G. (West Sussex, UK: John Wiley and Sons, Ltd.), 1–25.

[ref53] Zhang Y. Yang X. Liu D. Wang Z. (2020). Chinese college students’ parental attachment, peer attachment, and prosocial behaviors: the moderating role of respiratory sinus arrhythmia. Biol. Psychol. 150:107844. 10.1016/j.biopsycho.2020.107844, PMID: 31954187

